# Quantitative Genetic Analysis of Baboon Facial Variation Challenges a Classic Papionin Trait

**DOI:** 10.1093/iob/obag031

**Published:** 2026-06-22

**Authors:** A Thiebaut, C C Roseman, M C Mahaney, L J Hlusko

**Affiliations:** Centro Nacional de Investigación sobre la Evolución Humana, Paseo Sierra de Atapuerca 3, 09002 Burgos, Spain; Department of Evolution, Ecology, and Behavior, University of Illinois at Urbana-Champaign, 515 Morrill Hall, 505 S Goodwin Ave, Urbana, IL 61801, USA; Department of Human Genetics, University of Texas Rio Grande Valley, Brownsville, TX 78520, USA; South Texas Diabetes and Obesity Institute University of Texas Rio Grande Valley, Brownsville, TX 78520, USA; Centro Nacional de Investigación sobre la Evolución Humana, Paseo Sierra de Atapuerca 3, 09002 Burgos, Spain

## Abstract

The Linnaean family of monkeys that includes baboons, macaques, and mandrills (among others) is notably characterized by large, extended muzzles that vary in lateral nasal dorsum profile. In paleontological studies, this variation is referred to as the anteorbital drop (AOD). Here, we first devise a method to quantify this phylogenetic character. We then employ quantitative genetic analyses using a MCMCglmm model of facial variation in a captive, pedigreed baboon colony (*n* = 934), including admixed individuals derived from at least two *Papio* species, from the Southwest National Primate Research Center to assess the quantitative variation of the AOD, its genetic integration relative to other aspects of facial variation, and its relative evolvability. Specifically, we test two hypotheses to assess the appropriateness of the AOD as a way to characterize lateral nasal dorsum profile variation for paleontological studies. We find that the first hypothesis of limited variation within the population is rejected, as this admixed population demonstrates a remarkable degree of AOD variation spanning what is observed across papionin genera. The second hypothesis proposes that variation in the AOD is highly heritable and highly evolvable. Our results indicate that the AOD is highly heritable, but simultaneously less evolvable than other aspects of facial variation. Additionally, sex has little influence on AOD variation, in contrast to other facial measurements. These results challenge the use of AOD as a phylogenetic character, as it likely results from a broad range of non-sexually dimorphic processes that contribute to facial length. We caution against using AOD as a discrete character in phylogenetic analyses and invite further investigation of its biological significance. This study aims to illustrate how quantitative genetic approaches can probe the evolutionary relevance of traditional traits used in paleontology.

## Introduction

The foundation for cladistic, phylogenetic, and taxonomic analyses is the concept of morphological character variation (Hennig 1966; de Queiroz and Gauthier 1994). As methodological approaches have expanded to include evolutionary quantitative genetics and more nuanced geometric morphometric characterization of 3-dimensional morphology, we have the opportunity to deepen and challenge our understanding of the biological etiology that underlies character variation. In this study, we investigate the anatomical variation, quantitative genetic basis, and evolvability for one of the most defining characters (aka, traits) used in the taxonomic study of the Papionini, a group of primates that includes macaques, geladas, baboons, mangabeys, drills, mandrills, kipunji, and numerous extinct genera: the anteorbital drop (AOD). This feature characterizes the lateral nasal dorsum profile of the papionin face.

The papionins represent a highly diversified tribe of cercopithecoid monkeys who live across Africa, southern Europe, and Asia, and have a rich fossil record starting in the early Pliocene of Africa (Jablonski 1993; Hartwig 2002; Jablonski and Frost 2010; Rowe and Myers 2017). This clade’s evolutionary history is widely recognized to be complicated by extensive homoplasy, strong allometric effects, and conflicts between molecular and morphological signals (Szalay and Delson 1979; Singleton 2002; Frost et al. 2003; Perelman et al. 2011; Gilbert et al. 2013; Pozzi et al. 2014). Numerous studies have sought to clarify papionin evolution based on craniodental morphology so as to include fossil data (Collard and O’Higgins 2001; Williams et al. 2007; Gilbert and Grine 2010; Esteve-Altava et al. 2015; Hlusko et al.2016; Monson et al. 2017, 2020; Nishimura et al. 2019; Elton and Dunn 2020), and over-arching phylogenetic relationships have been proposed based on the cladistic analysis of this craniodental variation (Gilbert et al. 2013, 2018).

Facial prognathism is a highly variable feature across papionins, ranging from the moderately protruding faces of *Lophocebus* to the highly elongated, dog-like muzzles of *Mandrillus* and *Papio*. A consensus holds that prognathism is a derived trait that evolved convergently at least twice within the papionin clade (Benefit and McCrossin 1991, 1993; Collard and O’Higgins 2001; Harris 2000, 2002; Monson and Brasil 2025). Significant variation is also observed in facial and muzzle breadth across both extant and extinct papionins. Some taxa exhibit particularly broad skulls due to expanded zygomatic arches (e.g., *Theropithecus, Dinopithecus*) (Freedman 1957; Jablonski 1993; Frost et al. 2017; Monson et al. 2017; Brasil et al. 2025).

Among these facial traits, the AOD is one of the most important characters. For example, *Procercocebus* (formerly *Parapapio antiquus*) is distinguished, in part, from *Pliopapio, Papio, Mandrillus, Dinopithecus, Gorgopithecus*, and *Theropithecus* by the “absence” of an AOD (Gilbert 2007). Fossils of the extinct genus *Parapapio* are often identified and distinguished from *Papio* mainly based on their lack of an AOD, characterized by a lateral nasal dorsum profile that is straight or only slightly concave (Frost and Delson 2002; Heaton 2006; Jablonski and Frost 2010). Within *Papio, P. izodi* is considered more primitive (more *Parapapio-like*) due to its “variable” AOD, whereas *P. angusticeps* exhibits a “definitive” AOD, aligning more closely with its extant relatives (McKee 1993; Gilbert 2013; Gilbert et al. 2015). Freedman (1957) mentioned that the assignment of specimens from the site of Kroomdrai in South Africa to the *Papio* genus is largely based on the presence of a steep AOD of the nasal bones. *Mandrillus* have been described as similar to *Papio* and distinct from mangabeys and most *Macaca* species by their “marked” or “clear presence” of AOD (Frost and Alemseged 2007; Gilbert et al. 2015, 2018). Frost and Delson (2002) distinguished *Theropithecus* from other cercopithecoids by its “steeper” AOD, featuring a more pronounced angle and a longer vertical segment of the muzzle dorsum than *Papio*.

For decades, numerous concerns about how normal variation is incorporated into cladistic analyses have been raised (e.g., Trinkaus 1990; Stevens 1991; Asfaw et al. 1999; Lovejoy et al. 1999; Wood and Lieberman 2001; Collard and Wood 2007; González-José et al. 2008; Hlusko et al. 2004; Hlusko 2016; Hlusko et al. 2016, Monson et al. 2017; Brasil et al. 2025). From our perspective, two of these are of particular concern for the papionin face, and in particular, the AOD. The first is the conversion of continuous variation into discrete, qualitative characters. And second, is the assumption that, in the absence of knowledge about the underlying biological etiology, trait variation results from simplistic and independent biological mechanisms.

Despite the extensive reliance of the AOD in phylogenetic systematics and taxonomy, there is no clear definition as to what exactly the AOD is. Sometimes the AOD is described as the angle formed by the convexity between the sub-vertical upper face and the more or less sub-horizontal nasal dorsum of the muzzle, principally held by the nasal bone. Sometimes the AOD is described through related morphologies, like the degree of concavity/convexity of the whole lateral nasal dorsum profile, face or muzzle orientation, and facial flexion (Singleton 2002; Pugh and Gilbert 2018; Brasil et al. 2025). Additionally, these qualitative descriptions characterize the variation with imprecise descriptions. For example, AOD has been divided into the character states: “definitive, apparent, marked, preserved, weak, or absent” (Szalay and Delson 1979; Eck 1993; Gilbert 2007). In Gilbert (2013)’s cladistic analysis, the AOD was categorized into states: 0 = absent, 1 = intermediate, and 2 = present.

One approach to improve our phylogenetic and taxonomic analyses is to explore the biology of character variation at the micro-evolutionary scale. Numerous studies have demonstrated that the biological effects underlying population-level patterns of morphological variation are closely linked to macroevolutionary trends and constrain the rate and direction of morphological evolution (Lande 1979; Gould and Lewontin 1979; Cheverud 1982, 1984, 1995; Schluter 1996, 2000; Lynch and Walsh 1998; Shubin 2002; Leigh 2006, 2007; Carroll 2008; Marroig et al. 2009; Hlusko et al. 2011; Grieco et al. 2013; Voje et al. 2013; Hlusko et al. 2016; McGlothin et al. 2018; Rolland 2023). Quantitative genetic analyses offer a powerful framework for assessing pleiotropic effects and patterns of genetic integration, enabling biologists to investigate patterns of genetic variation underlying traits and their evolutionary relevance (Cheverud 1982, 1984; Sherwood et al. 2008; Hlusko et al. 2011; Jakobson and Jarosz 2020).

Here, we report on our effort to characterize the population-level variation and evolvability of the AOD through quantitative genetic analysis of facial variation in a captive, pedigreed, and admixed baboon colony. We adopt a conservative definition of AOD as the convexity of the nasal dorsum in the sagittal plane, between glabella and rhinion, taken from skulls oriented in the Frankfurt plane, and provide a quantitative method for characterizing this. Ours is the first attempt to characterize the quantitative genetic basis of papionin AOD variation, with the ultimate aim of informing and improving our understanding of papionin and, ultimately, primate craniofacial evolution.

Rather than a critique of earlier work, this study seeks to demonstrate how quantitative genetic approaches can help reassess the evolutionary significance of traditionally defined qualitative traits.

Our study tests two hypotheses derived from assumptions in the paleontological literature:

**H1**: Population-level variation in the AOD is restricted. To test this hypothesis, we developed a method for quantifying AOD from CT scans of skeletonized crania to investigate its variation within 934 related baboons from the Southwest National Primate Research Center (SNPRC).

**H2**:The AOD is highly heritable and evolvable, with minimal environmental effects to its phenotypic variance, and minimal shared genetic effects (pleiotropy) with its other traits.

To test this hypothesis, we conduct a series of quantitative genetic analyses on these same SNPRC baboons to characterize the genetic variance, covariance structure, and the evolvability of the AOD relative to other aspects of facial variation.

## Material and methods

### SNPRC baboon population

We collected facial landmarks from digitized skulls (CT scans) of *Papio sp.* from a pedigreed colony housed at the Southwest National Primate Research Center (SNPRC) in San Antonio, TX, USA. Previously, and in our earlier research on this population, the baboons in the SNPRC pedigreed colony were considered to be one species with representation from several subspecies (i.e., Cox et al. 2013; Hlusko et al. 2004). General consensus on the use of species concepts and taxonomic names has shifted over the years. Therefore, following this higher taxonomic elevation of population differences, the SNPRC baboon colony is now considered to be an admixed population of at least four *Papio* species *(P. anubis, P. cynocephalus, P. hamadryas*, and *P. ursinus*), and will therefore be referred to herein as *Papio sp.* (Mercuri et al. 2025). The colony’s breeding is controlled, enabling the creation of a complex pedigree of over 1365 animals, complete with detailed age, sex, and health records. The sub-sample used in this study spans over five generations within that pedigree. Among these individuals, 914 belong to a single extended subpedigree, while the remaining individuals are distributed across smaller family units. Apart from 26 singletons (unrelated individuals), these smaller units consist primarily of nuclear families. All pedigree data management and preparation were performed using the PEDSYS software (Dyke 1996). The full pedigree of 1365 animals was used to compute the relatedness structure in all models, while phenotypic data were available for a subset of individuals (*N* = 934), which constituted the analytical sample. The structure of the pedigrees is detailed in Table 1. Finally, the studied sample yielded a female-to-male sex ratio approximating 2.3:1, with *n* = 654 females and *n* = 280 males.

**Table 1:** Structure of the full pedigree and the phenotyped subset pedigree

					Proportion of relationships by degree			
	Number of individuals	Total number of pairs	Related-pair density	Close relationship density	1st degree	2nd degree	3rd degree	Mean kinship coefficient
**Full pedigree**	1365	930,930	1.18%	1.08%	17.10%	54.90%	20.10%	0.132
**Phenotyped individuals (subset of pedigree)**	934	435,711	2.48%	2.23%	6.50%	55.40%	27.40%	0.132

Summary of the number of individuals, **total number of pairwise, pairwise related pairs density** (corresponding to the proportion of related pairs), **close relationship density** (corresponding to the proportion of first- to third-degree related pairs among all possible pairwise comparisons), **distribution of relationship degrees** (first to third) among related pairs, and **mean kinship coefficient** among related pairs. The phenotyped subset represents the subset of individuals drawn from the full pedigree used in this study. The full pedigree was used to compute the relatedness structure in all models, while phenotypic data were available for a subset of individuals only.

### Phenotypes data methods

The skull collection used for CT scanning is currently housed at the University of Illinois Urbana-Champaign and comprises 985 individuals, all of which are publicly available for data collection through the image repository MorphoSource (Boyer et al. 2016). Recent work by Modesto-Mata et al. (2025) discussed some complexities associated with the use of this collection, and readers interested in working with these data are referred to that study for additional details. For this study, all specimens with abnormal facial morphology (e.g., deformed or broken) were excluded, as such conditions made it difficult to place landmarks accurately. In addition, individuals younger than 6 years were excluded. Individuals aged 6–10 years were retained but represent less than 10% of the final sample. The resulting dataset includes 934 individuals.

#### Data acquisition of linear facial traits

A total of 8 inter-landmark distances were acquired based on previous morphometric (Gilbert and Grine 2010; Monson et al. 2017) and cladistic/taxonomic analyses to characterize the face (Jablonsky and Leakey 2008; Gilbert 2013) (Table 2). Seven Landmarks (4 paired and 3 unpaired, Table 2) were placed on meshes segmented from CT scans, and inter-landmark distances were computed using Euclidean distances from their 3D coordinates. Data acquisition, including landmarking and distance calculations, was semi-automated via the ALPACA function from the SlicerMorph package (Fedorov et al. 2012; Rolfe et al. 2021) and R (R Core Team 2022). ALPACA duplicates a source landmark configuration and projects it onto a target mesh following mesh alignment, enabling automated landmark transfer.

**Table 2:** Landmarks definition and linear dimensions

**Landmarks**	
**No**	**Landmarks**
1	Glabella
2	Prosthion
3	Rhinion
4	Maxilla—premaxilla suture at the alveolar process
5	Most posterior point of the maxilla behind the third molar
6	Most anterior point of the upper zygomatic process
7	Zygomaticotemporale foramen
**Distances**	
**Landmarks pairs**	**Facial dimension**
1–2	Facial length
1–3	Nasal length
bi 4	Premaxilla breadth
bi 6	Mid-face breadth
2–4	Premaxilla lateral length
2–3	Premaxilla length
1–5	Facial heights
bi 7	Upper facial breadth

To ensure accuracy, this automated procedure was followed by a systematic verification and correction step: each specimen’s mesh and associated landmarks were visually inspected individually, and landmark positions were manually adjusted when necessary.

#### Data acquisition and analysis of the AOD

To characterize variation in AOD within the SNPRC population, we first explored its shape variation using two-dimensional General Procrustes Analysis (2D GPA) and principal components analysis (described in detail below). We then quantified AOD using a morphometric ratio (RC trait; see below) and confirmed that this metric captured the same pattern of variation by testing its correlation with the GPA-derived shape axes. This validated AOD measure was subsequently used for all quantitative analyses.

**Fig. 1:**
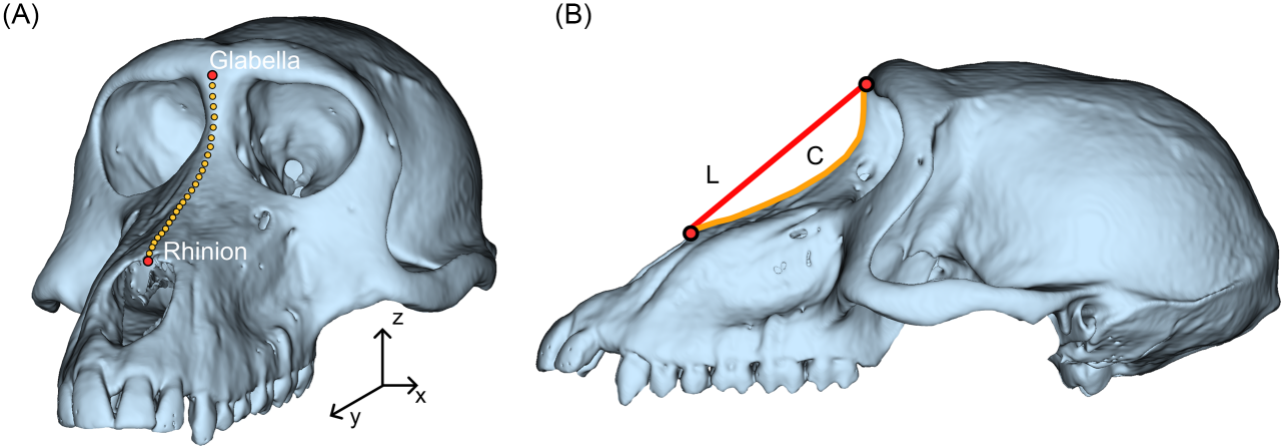
Two-step protocol for quantifying the anteorbital drop. (**A**) Geometric morphometric approach using 25 semi-landmarks placed along the nasal roof between glabella and rhinion. (**B**) Morphometric approach based on the computation of the RC index, defined as the ratio of the curved length (*C*) between glabella and rhinion to the straight-line distance (*L*) between the same points.

For the 2D GPA, the AOD was defined by 25 semi-landmarks placed between glabella and rhinion, following the nasal bone sagittal line (Fig. 1). Here, we measured AOD as the profile convexity between glabella and rhinion (or nasal dorsum). We selected this definition because it encompasses the range of alternative definitions previously applied to this trait. We avoided using the nasion, whose position varies markedly among taxa, to prevent interspecific bias when applying the same measurement protocol across multiple species. We recognize that using glabella may affect profile angle by the variable expression of a supraorbital torus; this potential influence is addressed in the discussion section. Semi-landmarks were generated by resampling a curve constrained to the mesh using 3D Slicer’s native “markups” module. Skulls were oriented laterally in 3D space, aligning their lateral profile with the y–z plane. The *x*-axis was then removed to perform 2D GPA on the y–z coordinates. GPA superimposition eliminates scale, translation, and rotation differences in landmark coordinates, enabling the extraction of variation patterns independent of allometry (Singleton 2002; Ito et al. 2014; Nishimura et al. 2019). We conducted a principal component analysis (PCA) on individual GPA coordinates to observe AOD’s major shape variation within the population (see Supplementary data for associated R script).

To translate the obtained principal AOD’s shape-variation into a quantitative trait suitable for populational statistics and quantitative genetic analysis, we measured the lateral relative curvature (RC) of the nasal dorsum as follows: length of the curve between the glabella and rhinion, divided by the linear distances between those two landmarks (Fig. 1). To avoid scaling issues and to allow comparison with other linear traits of this study, the RC ratio was multiplied by 100. We performed a Pearson correlation test to evaluate whether the RC trait captures the same variation as the GPA within the population. For all specimens, landmarks, curves, coordinates extraction, and distance measurements were acquired via a semi-automated pipeline in 3D Slicer, using Python scripts and the ALPACA function from the SlicerMorph package. As noted above, this procedure was followed by a secondary verification step, during which landmark placement and mesh condition were systematically inspected and corrected, prior to analysis. Statistical analyses were conducted in R, with GPA performed using the gpagen function from the geomorph package (Adams and Otárola-Castillo 2013).

### Quantitative genetic analysis

#### MCMCglmm model

We used an animal model framework to estimate the residual heritability (*h*²ᵣ) and evolvability (*e*) of the AOD, along with eight other facial linear traits, as well as the genetic correlations between trait pairs. All analyses were performed using the MCMCglmm package in R (Hadfield 2010). The animal model is a linear mixed-effects model that uses the complete pedigree structure to estimate genetic (co)variances among individuals. In its simplest form, it partitions the total phenotypic variance (*V*_p_) into additive genetic variance (*V*_a_) and residual variance (*V*_e_), the latter encompassing environmental and other unexplained effects. However, additional variance components (e.g., dominance, maternal, or shared environmental effects) can also be incorporated depending on the biological context and data structure. The known pedigree structure of the SNPRC baboon colony allows for accurate estimation of the additive genetic component, as relatedness among individuals informs the model. To reduce the risk of inflating heritability estimates due to confounding effects, we included sex as a fixed effect in the model (Wilson et al. 2008; Levine et al. 2021). Following standard biological assumptions for the distribution of quantitative traits, all traits were modeled under a Gaussian distribution. To run the multivariate animal models in MCMCglmm, prior distributions for the variance components must be specified. The package uses an inverse-Wishart distribution for the prior on the (co)variance matrices, parameterized by *V* (scale matrix) and *ν* (degrees of freedom). We compared two models with differing *ν* values, while assuming that phenotypic variance was equally partitioned between additive genetic and residual components (see Supplementary data for model description).

Model.11 used a more informative prior with *ν* = *p* + 2 (where *p* is the number of traits, here 9), following the default recommendation in MCMCglmm.

Model.9 applied a less informative prior with *ν* = *p*, providing looser constraints on the posterior.

To evaluate which prior better suited our data, we compared the models based on their deviance information criterion (DIC), convergence diagnostics, and the consistency of variance component estimates (*V*_a_) and associated 95% highest posterior density intervals (HPD). Both models were run using the same Markov chain Monte Carlo (MCMC) iterations parameters: 800,000 iterations, a burn-in of 250,000, and a thinning interval of 200. These settings were chosen to ensure model convergence, reduce autocorrelation, and yield sufficient effective sample sizes (Hadfield 2010; De Villemereuil 2020). Model diagnostics included Heidelberger and Welch’s convergence test, autocorrelation diagnostics, visual inspection of posterior density and trace plots, and verification that the effective sample size (ESS) exceeded 1000 for all parameters (Villemereuil 2012; Levine et al. 2021).

#### Quantitative genetic parameters acquisition

Estimates of variance components and genetic correlations were obtained from the posterior modes of the distributions, following standard Bayesian procedures. We computed 95% HPD intervals using the HPDinterval function from the coda package in *R* (Plummer et al. 2006). Heritability for each trait was calculated as the proportion of phenotypic variance attributable to additive genetic effects: $h_r^2 = \frac{{{V}_a}}{{{V}_p}}$ (Falconer and Mackay 1996). Uncertainty around h²ᵣ estimates was summarized using the 95% HPD interval. While heritability provides useful insight into the genetic basis of phenotypic variation, it is less informative for evolutionary comparisons because high values can arise from either low environmental variance or high genetic variance. To better assess the evolutionary potential of traits, we also calculated the coefficient of additive genetic variation, or evolvability (*e*), defined as: $= \frac{{{V}_a}}{{{{\mathrm{\mu }}}^2}}$; where *V*_a_ is the additive genetic variance and μ is the population mean of the trait. This parameter, computed from the posterior distributions of *V*_a_ and μ, allows for meaningful comparisons of evolutionary potential across traits (Houle 1992; Hansen and Houle 2004, 2008; Roseman et al. 2010). Trait differences in heritability and evolvability were evaluated using a Bayesian approach. For each pair of traits, we computed the posterior distribution of the difference (Δ) by subtracting posterior samples. We summarized each contrast using the posterior mode and 95% HPD interval. Difference is considered credibly different from zero when its 95% HPD interval excludes zero.

Genetic correlations between trait pairs were obtained by applying the var2cor function in *R* to the posterior mode of the additive genetic variance–covariance matrix.

We tested the effect of sex on trait phenotypic variation by computing the proportion of variance due to fixed effects (*R*²) following Nakagawa and Schielzeth (2013). This corresponds to the ratio between the variance explained by the fixed effect and the total phenotypic variance of the trait, where the variance of sex as a fixed effect is computed as ${V}_{sex} = {\beta }^2_{sex}\ p( {1 - p} )$, with *p* being the proportion of females (variance of a Bernoulli(*p*) distribution).

## Results

### General procrustes analysis of the AOD

**Fig. 2:**
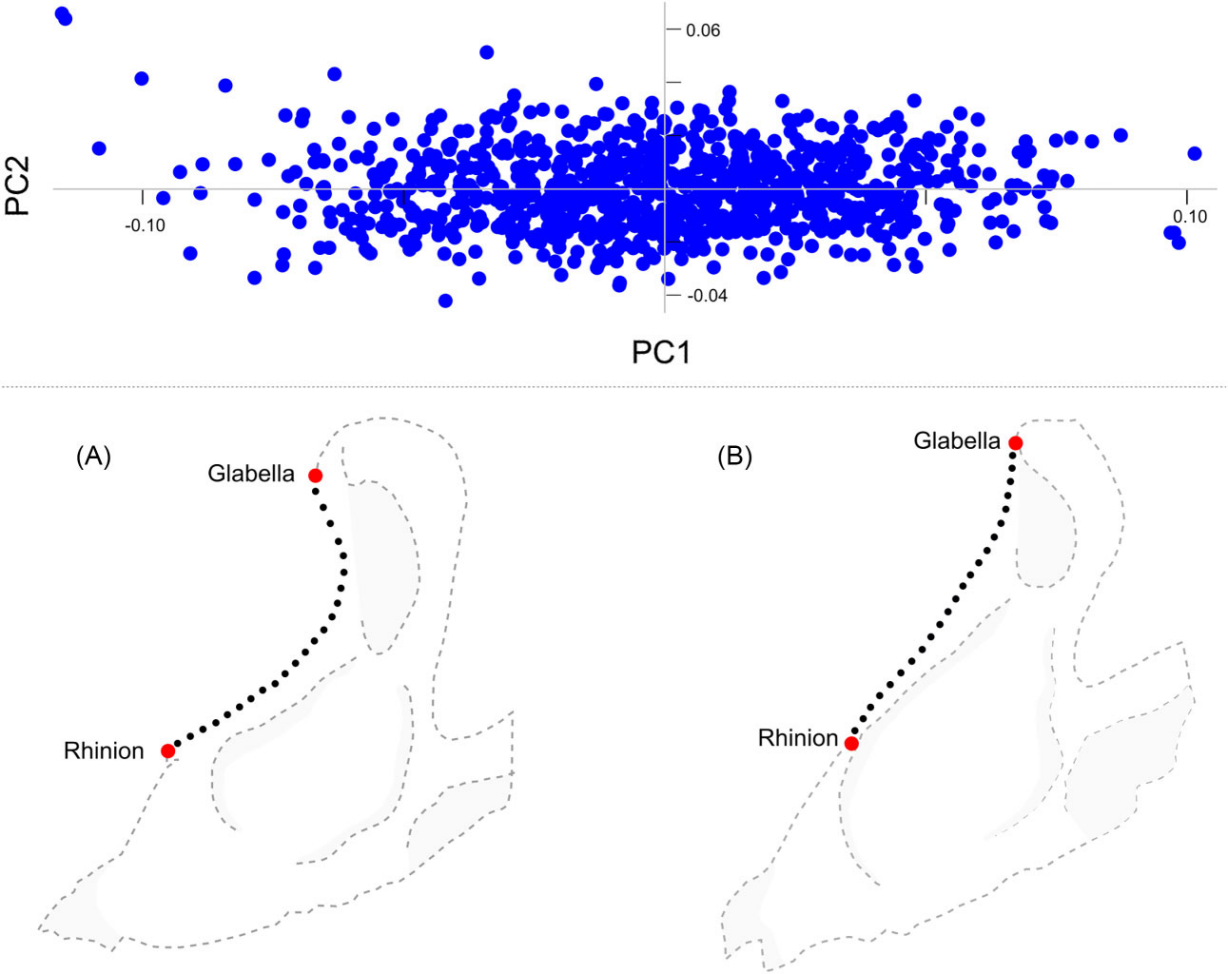
Principal Component Analysis on Procrustes-aligned coordinates and associated Shape deformation of the anteorbital drop along the first principal component (PC1). Panel (**A**) shows a low PC1 value, associated with a curved nasal dorsum profile. Panel (**B**) shows a high PC1 value, associated with a straight nasal dorsum profile. Skull silhouettes are overlaid for visualization and oriented in the Frankfurt plane.

We found a considerable amount of variation in the AOD within the SNPRC baboons’ population. PCA on GPA coordinates shows that the two first PC accounted for >85% of the variation in the AOD (lateral facial profile), with PC1 summarizing 75.18% and the PC2, 13.18% of the variation. The distribution along PC1 is continuous, fairly symmetric (skewness = −0.16), and follows a normal distribution (Shapiro–Wilk test: *W* = 0.99, *P* = 0.09). Shape variation along PC1 primarily reflects the convexity degree of the glabella-to-rhinion transition, the anatomical region referred to as the AOD (Fig. 2). Individuals with lower scores exhibit a sloped nasal dorsum profile, with some showing pronounced angulation (i.e., a prominent AOD), whereas higher scores correspond to a straighter nasal dorsum profile with minimal angulation (i.e., an absent AOD; Fig. 2). PC2 distribution is slightly asymmetric (skewness = 0.32) and deviates from normality (Shapiro–Wilk test: *W* = 0.99, *P* = 5.87e-05). Shape variation along PC2 is subtle, primarily reflecting minor topological differences in curve morphology.

### Relative curvature of the profile and GPA

Our approach for quantifying AOD is effective. The relative curvature of the nasal dorsum (RC) shows a strong correlation with scores of PC1 (Pearson’s product-moment correlation, cor = −0.88; *P*-value = <2.2e-16). The negative correlation indicates that individuals with high RC values have the lowest PC1 scores, exhibiting a more convex profile, whereas lower RC values correspond to individuals with a straighter glabella–rhinion transition.

**Fig. 3:**
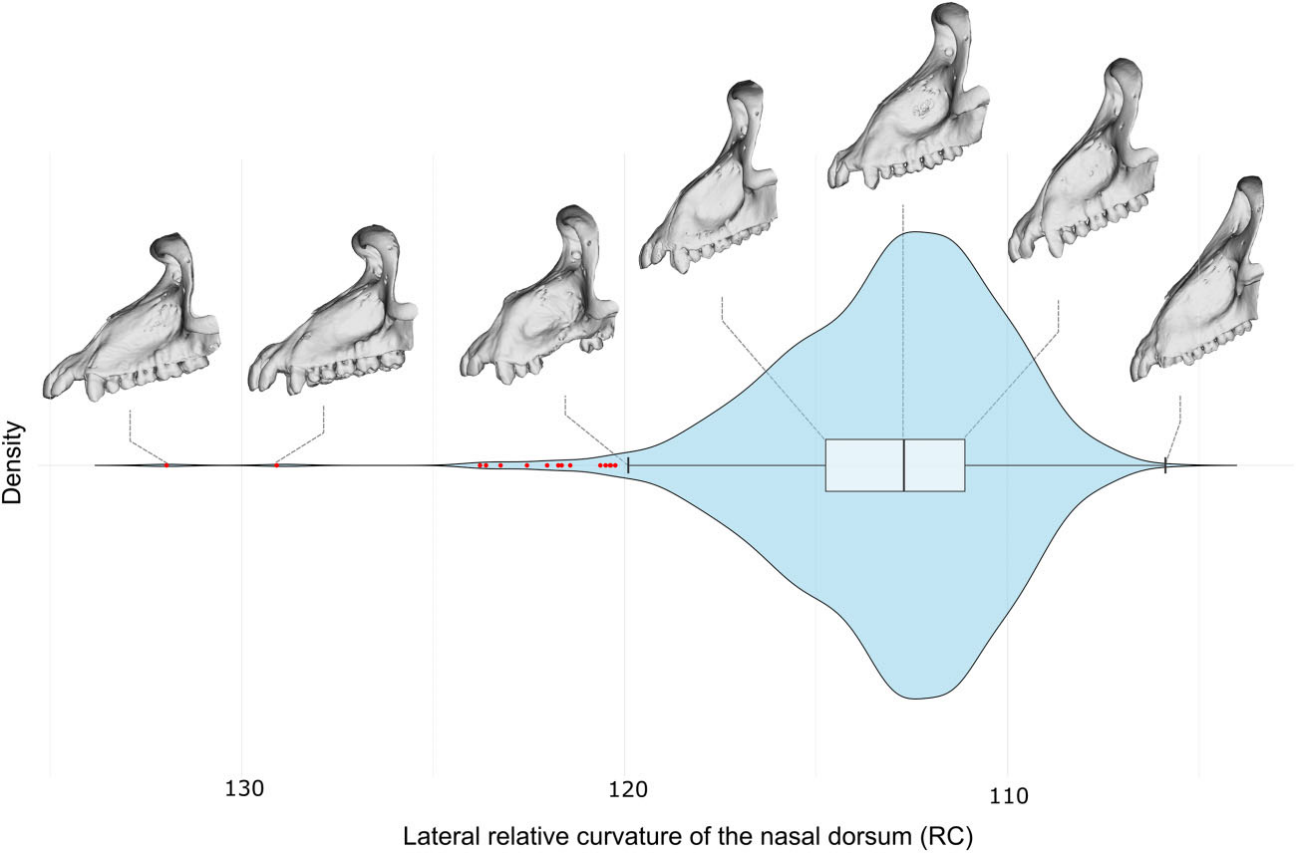
Distribution of relative curvature of the nasal dorsum (RC) in SNPRC baboons. Boxplots show the mean, quartiles, and the range of the normal distribution, with red points indicating statistical outliers. Representative *Papio hamadryas* meshes corresponding to key positions along the distribution are displayed. All skulls are oriented in the Frankfurt plane; the cranial vault and cranial base are omitted for clarity.

The RC trait shows a continuous and normal distribution. Visual inspection of individuals representing different RC values along this distribution (mean, first quartile, boundaries of the normal range, and outliers) reveals marked morphological variation in the nasal dorsum profile within the SNPRC *Papio sp.* population (Fig. 3). Thus, the RC trait effectively captures variation in nasal dorsum profile convexity, referred to here as the AOD. Accordingly, we will use the term AOD throughout the remainder of the paper to refer to this measurement.

### Quantitative genetic parameter estimates

#### Models sensitivity and selection

The two different prior settings tested for the MCMCglmm models had negligible impact on parameter estimates. Genetic variance components estimates were nearly identical across priors, with overlapping 95% HPD for each estimate (Supplementary Table S.1), demonstrating robustness of the results to prior choice. Both models (*ν* = 9 and *ν* = 11) return comfortable effective sample sizes (all parameters *N*_eff_ > 2500, many exceeding 3000), showed good mixing (lag-1 autocorrelation <0.1), and passed Heidelberger–Welch diagnostics (0% failures for stationarity; <0.5% for half-width). Model 9, however, exhibited a markedly lower DIC than Model 11 (37966.8 vs. 38215.5; ΔDIC~248), indicating a substantially better fit to the data. We therefore retained Model 9 as our main analysis, and all results reported below are based on this specification, using the weak prior (*ν*=9) that assumes equal partitioning of total phenotypic variance between additive genetic and residual components.

#### Heritability, evolvability, and sex effects (Table 3)

The posterior modes of narrow-sense heritability estimates across traits ranged from 0.35 to 0.66, with substantial overlap in the 95% HPD intervals for most traits. Exactly 50% of pairwise traits showed credible differences in heritability (Supplementary Table S.2). The AOD exhibited the highest heritability estimate, but did not differ credibly from other highly heritable traits (*h*²ᵣ > 0.5), including facial length, nasal length, facial height, upper facial breadth, and premaxilla length. Credible differences were detected only between AOD and premaxilla breadth, mid-facial height, and premaxilla lateral length.

The posterior modes of evolvability and their 95% HPD intervals reveal a marked heterogeneity among traits. Approximately 75% of pairwise traits showed credible differences in evolvability (Supplementary Table S.3). The AOD differed credibly from all other traits, exhibiting the lowest evolvability. In contrast, premaxilla length, facial length, and nasal length showed the highest evolvability estimates, whereas upper facial breadth, mid-facial breadth, and facial heights occupied the lower end of the evolvability spectrum.

The posterior modes of *R*^2^ estimates, representing the proportion of total phenotypic variance explained by the fixed effect sex, vary substantially across traits, ranging from 12 to 75%. AOD shows the lowest value, credibly different from all premaxilla measurements, exhibiting intermediate values ranging from 23 to 40%. In contrast, the highest values for the effect from sex (i.e., face length, breadth, and height) range from 68 to 75% and do not show credible differences, as they all overlap within their 95% HPD.

### Trait pairwise analysis

**Fig. 4:**
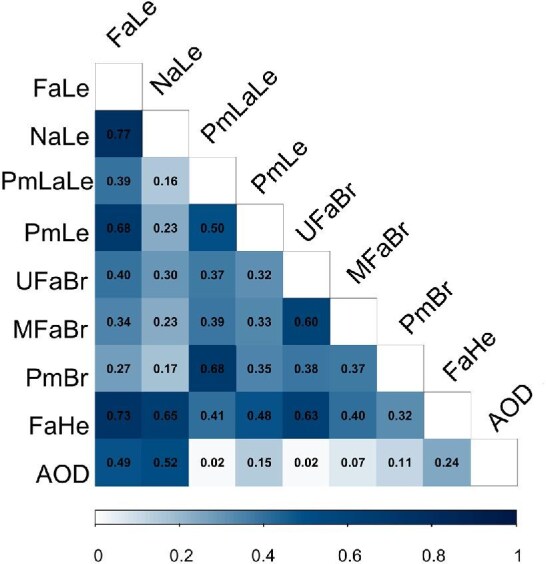
Matrix of genetic correlations between pairs of quantitative traits (ρ_G_) based on the genetic correlation matrix obtained from the posterior mode of the variance–covariance matrix. ρ_g_ are expressed in absolute values, see Table 3. for directions of the correlations. Abbreviations: FaLe, facial length; NaLe, Nasal length; PmLaLe, premaxilla lateral length; PmLe, premaxilla length; UFaBr, upper facial breadth; MFaBr, mid-facial breadth; PmBr, premaxilla breadth; and FaHe, facial height.

Summary of paired-traits genetic correlations are presented as *ρ*_G_ matrices of correlations in Table 4 and Fig. 4.

Following the classification proposed by Hlusko et al. (2011), we interpret genetic correlations (ρ_g_) as follows:

- for ρ_g_ = 0 no pleiotropy.
- for ρ_g_ = 0−0.69: incomplete pleiotropy.
- for ρ_g_ > 0.69: incomplete pleiotropy but strong.
- for ρ_g_ = 1: complete pleiotropy.

Overall, no consistent pattern of genetic integration (correlation) emerges across breadth, length, and height measurements. Additionally, no two dimensions show a genetic correlation of one. However, two trait pairs do exhibit high genetic correlations (ρ_g_ > 0.69), indicating incomplete pleiotropy (i.e., facial length with facial height or nasal height). Most trait pairs display genetic correlations ranging from 0.15 to 0.68, while some are close to zero (e.g., AOD, premaxilla lateral length, and facial upper breadth: ρ_g_ = 0.02).

Some measurements display a strongly integrated pattern. Facial length and height show a high number of incomplete pleiotropy relationships with other dimensions, with 50% of their correlations exceeding ρ_g_ ≈ 0.5. In contrast, AOD only shows two correlations around ρ_g_ ≈ 0.5 (i.e., with facial and nasal length) while other correlations do not exceed ρ_g_ ≈ 0.25. Although the strong correlation between AOD and nasal length may appear interesting at first glance, it is not surprising because nasal length directly contributes to the calculation of AOD; therefore, they are genetically correlated by definition. Premaxillary measurements exhibit a specific pattern of genetic correlation as most of the ρ_g_ values with other linear distances rarely exceed 0.40 (except for premaxilla length and facial length), but increase toward ρ_g_ = 0.5–0.68 when paired with other premaxilla traits.

## Discussion

The aim of this study was to investigate the phenotypic variation and quantitative genetic basis of the AOD, a facial feature extensively used in the study of papionin evolution. To explore the evolutionary potential of this morphological feature, we tested its phenotypic variability at the population level in *Papio sp.* Using the same population, we then estimated the heritability and evolvability of a quantitative proxy of this trait in relation to other aspects of facial variation.

### Test of hypothesis 1: AOD phenotypic variation

AOD, as estimated here by the convexity of the nasal dorsum between glabella and rhinion displays substantial variability within the SNPRC population, ranging from straight to highly curved conformation (Figs. 2 and 3). This variation is largely continuous as expected for a quantitative trait in biological populations (Lynch and Walsh 1998; Falconer and Mackay 2022), which contrasts with previously defined discrete variation in the AOD (Gilbert et al. 2013). AOD as described by RC variation, exhibits low relative and absolute dispersion, suggesting that despite the high observed range of variation, most individuals display a similar degree of AOD, as supported by the subtler differences between individuals at the quartile boundaries (Fig. 3). Nevertheless, the within-normal distribution range reveals considerable variation, from concave nasal dorsum to straight, downward-flexed conformation. Extreme convex morphologies are held by a few outlier individuals (all females) and are largely influenced by a pronounced supraorbital torus. In many cases, the nasion is embedded within this bony structure, raising questions about the potential bias introduced by the supraorbital torus in qualitative assessments of the AOD when evaluated from the nasion.

**Fig. 5:**
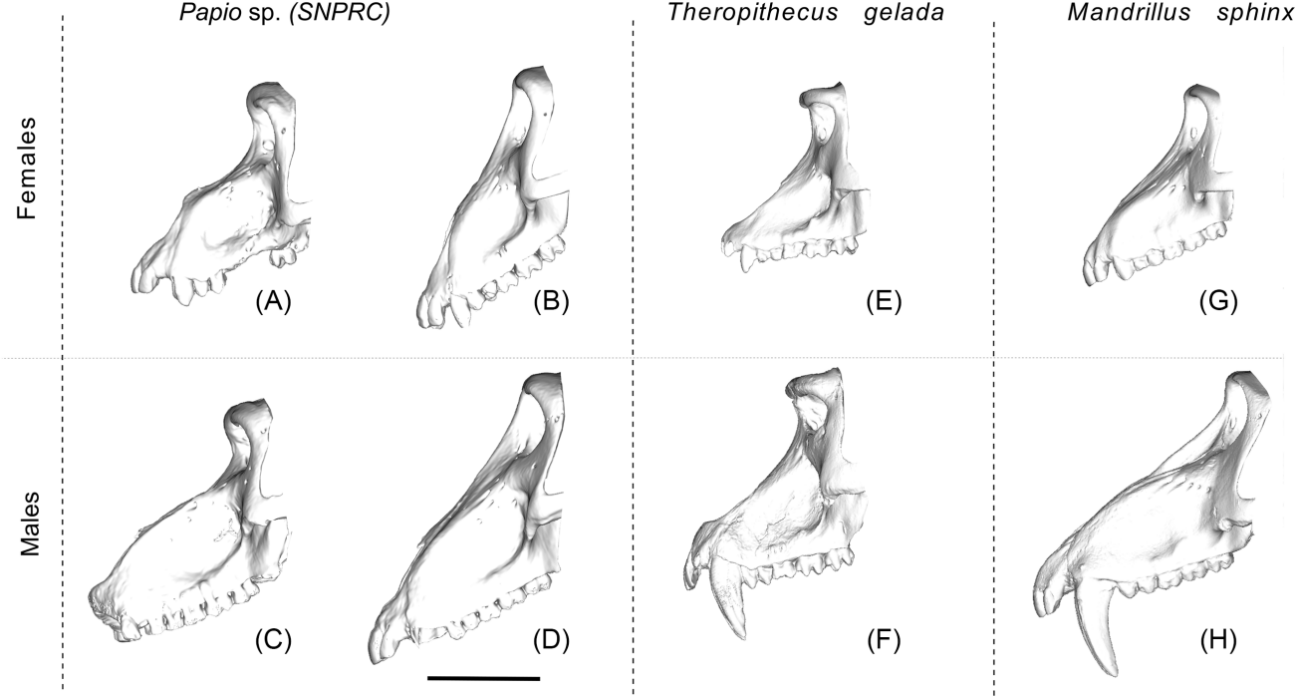
Comparative visualization of anteorbital drop variation in SNPRC baboons, *Theropithecus*, and *Mandrillus*, including both males and females. The reference SNPRC *Papio* sp. specimens correspond to the extreme values within the normal distribution of the anteorbital (AOD) drop trait (see text), from higher values (left) to lower values (right). All skulls are oriented in the Frankfurt plane. The cranial vault and cranial base are omitted for clarity. Scale bar: 5 cm. (**A**–**H**) ID reference of each specimen. (**A**) W193; (**B**) W313; (**C**) W501; (**D**) W373; (**E**) USNM 354990; (**F**) USNM 305107; (**G**) USNM 598493; and (**H**) USNM 598494.

When qualitative comparisons are performed using the same approach traditionally applied to assess the AOD in *T. gelada* and *M. sphinx*, the extensive variation observed within the SNPRC baboon population complicates trait evaluation. The distinction between *Papio, Theropithecus*, and *Mandrillus* appears less clear than traditionally assumed (Fig. 5). The “*Theropithecus* conformation” does not differ markedly from some variants observed within the SNPRC colony, as illustrated in Fig. 5, for both males and females. In contrast, *M. sphinx* exhibits a distinct shape relative to the admixed *Papio* population examined here, with a different origination of the nasal bone curvature.

These results challenge the notion of a “clearly defined” or “present” AOD in *Papio* as previously described (Frost and Alemseged 2007; Gilbert 2013; Gilbert et al. 2015, 2018), and suggest that this state assigned to *Papio* may be ambiguous at the population level. Our findings echo those of Nishimura et al. (2019) who, in an analysis of species-level variation in the AOD, reported that variation among *Papio, Mandrillus*, and *Theropithecus* might not be as clear as commonly declared, based on a geomorphometric approach. For a trait to hold phylogenetic significance, it must differ consistently between taxa and show little variation within populations and species. This criterion is not met by AOD, as it shows both high within-population variability and limited interspecific differentiation (Farris 1966; Lieberman 1999; Wiens 2001; Strait and Grine 2004; Williams and Ebach 2020) (Figs. 3 and 5).

**Fig. 6:**
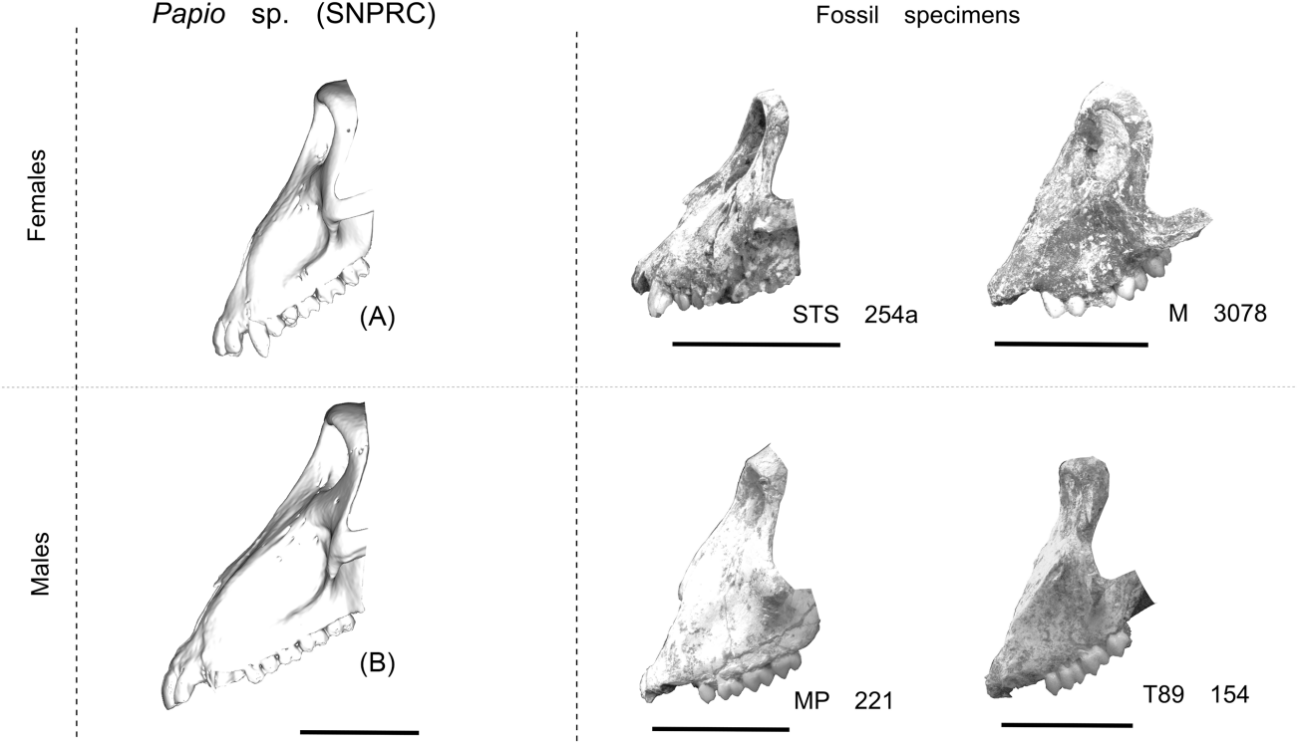
Comparative visualization of anteorbital drop variation in SNPRC baboons, *Parapapio* sp. (STS 254a and MP 221), and *Procercocebus antiquus* (M 3078 and T89 154), including both males and females. The reference SNPRC *Papio* sp. individuals correspond to the highest values within the normal distribution of the anteorbital drop trait (see text). All skulls are oriented in the Frankfurt plane. Scales are not consistent between specimens to facilitate visual comparison, as some specimens are significantly smaller than extant *Papio* sp. Scale bar: 5 cm. The cranial vault and cranial base are omitted for clarity. (**A**) W313; (**B**) W373.

This ambiguity also bears on fossil taxonomic diagnoses. Gilbert (2013) reported the exclusion of *Papio izodi* from the *Papio* clade based on the presence of primitive features, including a “variable” AOD. They noted that including *P. izodi* within the *Papio* clade would broaden the range of variation observed for these characters: a possibility that is not inconsistent with our findings on AOD’s *Papio* population variation amplitude. The genus *Parapapio*, traditionally described as distinct from *Papio* based on the absence of an AOD (or straighter nasal dorsum profile), does not show such clear differences when compared to straight-conformation specimens from the SNPRC population (Fig. 6). *Parapapio* fossil specimens STS 254a and MP 221 both exhibit qualitative conformation comparable to those of specimens from the SNPRC *Papio* population. A similar pattern is observed for *Procercocebus antiquus* (see specimens M 3078 and T89-154). Although the absence of an AOD and the presence of a straight nasal dorsum profile has been proposed as one of the diagnostic characters distinguishing *Procercocebus* from large papionins, this distinction appears less evident when viewed in the context of variation in SNPRC *Papio* population. In particular, the female fossil specimen (M 3078) falls well within the range of variation observed in the SNPRC population, whereas the male specimen (T89-154) shows a more pronounced divergence.

### Test of hypothesis 2: Quantitative genetic and evolutionary potential of the AOD

Heritability estimates obtained from the MCMCglmm model show only moderate variation across facial traits, suggesting a generally uniform level of additive genetic influence within the facial module (Esteve-Altava et al. 2015). The AOD exhibits a relatively high heritability (*h*²_r_ = 0.66), yet this value does not credibly differ from those of other traits, as indicated by largely overlapping 95% HPD intervals (e.g., facial and nasal length, facial height, and upper and mid-breadth). These results are consistent with Roseman et al. (2010), who found no evidence for greater heritability in any particular cranial region or trait in this same admixed population. Moreover, AOD exhibits significantly lower evolvability estimates than other traits, indicating a limited evolutionary potential and a reduced expected response to directional selection (Houle 1992; Hansen and Houle 2008; Roseman et al. 2010). The marked disparity in evolvability estimates across traits, despite relatively homogeneous heritability, supports the use of evolvability as a more informative metric of evolutionary potential (Houle 1992; Hansen and Houle 2004, 2008). In particular, although AOD appears highly heritable, its low evolvability index may reflect stabilizing selection that limits genetic variation available for directional selection within the population (Roseman et al. 2010). This low evolvability index suggests that even though the trait is highly heritable and varies considerably within the SNPRC population, this variation is not as responsive to selective pressures as are other aspects of facial variation.

Also, relative to other traits, AOD appears to be fairly genetically independent as its pattern of integration reveals only one substantial genetic correlation (ρ_g_ ≈ 0.5; facial length). Although poorly integrated, the pleiotropic relationship between face length and AOD in this population warrants further investigation since facial length (as a proxy for prognathism) is a trait extensively used in papionin’s taxonomy and phylogeny (Jablonski and Leakey 2008; Gilbert and Grine 2010; Gilbert 2013; Frost et al. 2017; Monson et al. 2017; Monson 2020). Given that patterns of genetic integration constrain phenotypic evolution and shape macroevolution dynamics (Cheverud, 1989; Marroig and Cheverud 2001; Grieco et al. 2013), we hypothesize that the pleiotropic association between facial length and AOD observed in the SNPRC admixed population may have persisted throughout papionin evolutionary history. This would suggest that inter-individual variation in AOD may partly reflect differences in facial length, potentially biasing evolutionary interpretations when considered independent (e.g., cladistic matrices, taxonomic identification).

Visual inspection of Fig. 3 shows how this reported pleiotropic constraint shapes the phenotypic variation of the muzzle in this population. As the muzzle elongates (face length), it undergoes ventral flexion, the facial tip shifts downward, the profile angle increases, and the nasal bone straightens from glabella to rhinion. O’Higgins and Collard (2002) reported comparable shape changes between males and females in papionins, with males showing a more inferior and anterior positioning of prosthion and an increased subnasal height. Similarly, Eck (1993), reported marked variation in AOD steepness within *Theropithecus*, influenced by sex, with females exhibiting a steeper drop than adult males. However, in our analysis, AOD variation is not structured by sexual dimorphism, the difference between males and females expressed as the regression coefficient (β_sex_), is less than 2% of the population mean. Our, MCMCglmm model estimates that sex accounts for *R*² = 12% of the variance. This contrasts with a large majority of traits for which sexual dimorphism contributes greatly to their phenotypic population variation (*R*² >40–70%) (Table 3), as it has been reported for most of the facial traits in cercopithecoids (Leigh and Cheverud 1991; Plavcan 2001; O’Higgins and Collard 2002). This pattern suggests that AOD variation is less likely to be driven by sexual dimorphism but may instead reflect pleiotropic associations with facial length and related traits involved in the downward shift of the face, consistent with developmental constraints (Leigh and Cheverud 1991; Plavcan 2003; Roseman et al. 2010). We therefore propose that these patterns are better explained by pleiotropic effects involving facial length, which is itself strongly influenced by body size (Joganic et al. 2018; Joganic and Heuzé 2019), potentially confounding apparent signals of sexual dimorphism (Plavcan 2001, 2003).

**Table 3:** Posterior mode estimates (with 95% HPD intervals) of residual heritability (h²ᵣ), evolvability (e), and the proportion of phenotypic variance explained by sex (*R*²) for the AOD and eight linear facial traits in the SNPRC *Papio* sp. population, based on MCMCglmm models

	*h*²ᵣ		*e*		Proportion of variance due to sex (*R*^2^)	
Trait	Mode	95% HPD	Mode	95% HPD	Mode	95% HPD
Facial length	0.64	0.53–0.74	0.0030	0.0025–0.0039	0.74	0.71–0.76
Nasal length	0.65	0.56–0.75	0.0048	0.0038–0.0058	0.75	0.72–0.77
Premaxilla lateral length	0.35	0.22–0.45	0.0029	0.0019–0.0044	0.29	0.23–0.33
Premaxilla length	0.51	0.41–0.63	0.0052	0.0039–0.0065	0.40	0.36–0.45
Upper facial breadth	0.58	0.48–0.66	0.0013	0.0011–0.0016	0.75	0.73–0.77
Mid-face breadth	0.48	0.38–0.57	0.0012	0.0009–0.0015	0.74	0.72–0.76
Premaxilla breadth	0.39	0.27–0.51	0.0028	0.0020–0.0040	0.23	0.18–0.27
Facial heights	0.61	0.50–0.69	0.0022	0.0017–0.0028	0.68	0.65–0.71
Anteorbital drop	0.66	0.54–0.77	0.0004	0.0003–0.0005	0.12	0.09–0.16

**Table 4:** Posterior mode of the genetic correlation matrix among studied traits, derived from the posterior mode of the variance/covariance matrix estimated using the MCMCglmm model

	FaLe	NaLe	PmLaLe	PmLe	UFaPBr	MFaBr	PmBr	FaHe
NaLe	0.77	X	|	|	|	|	|	|
PmLaLe	0.39	0.16	X	|	|	|	|	|
PmLe	0.68	0.23	0.50	X	|	|	|	|
FaUPBr	0.40	0.30	0.37	0.32	X	|	|	|
MFaBr	0.34	0.23	0.39	0.33	0.60	X	|	|
PmBr	0.27	0.17	0.68	0.35	0.38	0.37	X	|
FaHe	0.73	0.65	0.41	0.48	0.63	0.40	0.32	X
AOD	−0.49	−0.52	−0.02	−0.15	0.02	0.07	0.11	−0.24

*Note*. Abbreviations: FaLe, facial length; NaLe, nasal length; PmLaLe, premaxilla lateral length; PmLe, premaxilla length; UFaBr, upper facial breadth; MFaBr, mid-facial breadth; PmBr, premaxilla breadth; and FaHe, facial height.

## Conclusion

Our morphometric analyses demonstrate substantial continuous phenotypic variation in the AOD within this admixed baboon population, challenging the notion that the trait exhibits restricted within-population variation. Moreover, qualitative comparisons with other papionin taxa (*Theropithecus, Mandrillus*, and *Parapapio*) suggest that interspecific differences in the AOD, when evaluated against the range of variation observed in this population, may be less clearly defined than previously proposed. Our quantitative genetic analyses indicate that AOD variation in this admixed population shows little genetic integration with facial breadth and size variation in the premaxilla, is not highly sexually dimorphic, but does have pleiotropic effects with facial and nasal length. Therefore, when the genetic integration with facial and nasal length is included in a phylogenetic analysis, AOD may not be a useful character. The low evolvability of AOD compared to the other quantitative measurements in our analysis is curious. We interpret this combination of high heritability but low evolvability as evidence that AOD is poorly responsive to directional selection at the microevolutionary scale, and that the higher taxonomic interpretation of AOD variation is more likely the result of macroevolutionary forces acting on facial length.

It is important to note, however, a potential caveat to our analyses. Much like the tendency of wild baboons to mate freely and often in the contact zones between populations/species (Bergman et al. 2008; Jolly et al. 2011), the SNPRC *Papio* pedigreed population has substantial contributions from at least two species (*P. anubis* and *P. cynocephalus*), and possibly additional input from *P. hamadryas* and *P. ursinus* (Mercuri et al. 2025). Over the past decades, the six morphologically distinct and geographically parapatric populations have been elevated from subspecies to species-level distinction within the genus *Papio* (Rogers et al. 2019; Kopp et al. 2023). But even though *P. anubis* and *P. cynocephalus* are estimated to have diverged around 1 Ma, and possibly more than 2 Ma (Rogers et al. 2019; Kopp et al. 2023), they still mate at contact zones (such as Amboseli, Kenya; Alberts and Altmann 2001). Consequently, deep population structure and significant admixture contribute significantly to baboon genomic and phenotypic variation (Guevara and Steiper 2014; Zinner et al. 2018; Walker et al. 2019; Taylor et al. 2023).

Previous studies have reported the phenotypic consequences of hybridization in this SNPRC baboon population: hybrid individuals are reported to exhibit cranial and dental phenotypes that are heterotic relative to parental populations, and sometimes with morphological abnormality, such as supernumerary teeth or dysmorphology of the cranial vault (Ackermann et al. 2006; Ackermann et al. 2014). In another papionin, macaques, hybridization appears to have very little effect on pelvic or mandibular shape, or body size (Buck et al. 2021, 2025; Brenner et al. 2026), but does seem to influence the development of skull allometry (Ito et al. 2024). Therefore, it is possible that the structure of admixture in the SNPRC pedigreed baboon colony has a unique influence on the genetic variance structure of AOD variation compared to other cercopithecids, and as such, caution is warranted when interpreting the evolutionary implications of our results.

Given that the SNPRC population has been shown to constitute a relevant and informative model system for investigating evolutionary patterns in papionins and, more broadly, cercopithecoids (Roseman et al. 2010; Hlusko et al. 2011; Grieco et al. 2013; Hlusko et al. 2016), we conclude that our study provides an important first step toward a better characterization of qualitative traits in papionin cranial evolution. Further work, particularly at larger scales and/or in natural populations, will be necessary to assess the generality of these patterns. For now, we caution against interpreting AOD as a discrete “trait” and invite a reassessment of its biological significance.

## Supplementary Material

obag031_Supplemental_File

## Data Availability

Raw data and analysis code are available from the corresponding author upon reasonable request.
