# Supplementary material for: Quantitative Genetic Analysis of Baboon Facial Variation Challenges a Classic Papionin Trait
Source: Integr Org Biol. 2026 Jun 22;8(1):obag031. doi: 10.1093/iob/obag031 (PMC13334917; doi:10.1093/iob/obag031)
Supplement: obag031_Supplemental_File [file obag031_supplemental_file.pdf]

Supplementary Table S.1: Comparison of additive genetic variance estimates across competing models (Model 9 and Model 11) using posterior modes and 95% HPD intervals.

Trait	model 9		model 11		Overlap
	mode	95% HPD	mode	95% HPD	
Facial length	33.98	27.66 - 44.09	35.83	27.93 - 43.51	YES
Nasal length	18.46	14.64 - 22.33	18.31	14.21 - 21.65	YES
Premaxilla lateral length	1.46	0.98 - 2.26	1.53	1.10 - 2.35	YES
Premaxilla length	10.80	8.02 - 13.61	9.97	7.94 - 13.27	YES
Upper facial breadth	8.02	6.64 - 10.23	8.43	6.63 - 10.24	YES
Mid-face breadth	10.28	8.16 - 13.41	10.32	8.30 - 13.55	YES
Premaxilla breadth	3.42	2.41 - 4.92	3.70	2.41 - 5.03	YES
Facial heights	12.05	9.56 - 15.10	12.06	9.40 - 15.01	YES
Anteorbital drop	4.76	3.69 - 6.01	4.83	3.61 - 5.86	YES

Supplementary Table S.2. Posterior modes and 95% highest posterior density (HPD) intervals of pairwise differences in narrow-sense heritability (h^2) among traits. For each pair of traits, we computed the posterior distribution of the difference by subtracting posterior samples of narrow-sense heritability obtained from the fitted Bayesian mixed model (model 9). Each difference distribution is summarized by its posterior mode and 95% highest posterior density (HPD) interval. A difference is considered credibly different from zero when its 95% HPD interval does not include zero. Abbreviations: FaLe – Facial length; NaLe – Nasal length; PmLaLe – Premaxilla lateral length; PmLe – Premaxilla length; UFaBr – Upper facial breadth; MFaBr – Mid-facial breadth; PmBr – Premaxilla breadth; FaHe – Facial height.

		Posterior distribution of the difference		
**Trait 1**	**Trait 2**	**mode**	**95% HPD**	**Include 0**
FaLe	NaLe	-0.01	[-0.12 ; 0.06]	YES
FaLe	PmLaLe	0.32	[0.15 ; 0.43]	NO
FaLe	PmLe	0.14	[0.02 ; 0.22]	NO
FaLe	UFaBr	0.07	[-0.06 ; 0.19]	YES
FaLe	MFaBr	0.17	[0.02 ; 0.28]	NO
FaLe	PmBr	0.24	[0.10 ; 0.39]	NO
FaLe	FaHe	0.04	[-0.06 ; 0.13]	YES
FaLe	AOD	-0.02	[-0.15 ; 0.13]	YES
NaLe	PmLaLe	0.35	[0.16 ; 0.46]	NO
NaLe	PmLe	0.15	[0.00 ; 0.28]	YES
NaLe	UFaBr	0.1	[-0.05 ; 0.21]	YES
NaLe	MFaBr	0.18	[0.04 ; 0.31]	NO
NaLe	PmBr	0.29	[0.12 ; 0.42]	NO
NaLe	FaHe	0.07	[-0.04 ; 0.17]	YES
NaLe	AOD	0	[-0.11 ; 0.13]	YES
PmLaLe	PmLe	-0.17	[-0.31 ; -0.05]	NO
PmLaLe	UFaBr	-0.23	[-0.38 ; -0.10]	NO
PmLaLe	MFaBr	-0.14	[-0.28 ; -0.01]	NO
PmLaLe	PmBr	-0.04	[-0.14 ; 0.05]	NO
PmLaLe	FaHe	-0.23	[-0.42 ; -0.12]	NO
PmLaLe	AOD	-0.35	[-0.48 ; -0.16]	NO
PmLe	UFaBr	-0.06	[-0.20 ; 0.07]	YES
PmLe	MFaBr	0.01	[-0.10 ; 0.17]	YES
PmLe	PmBr	0.1	[-0.01 ; 0.28]	YES
PmLe	FaHe	-0.08	[-0.20 ; 0.06]	YES
PmLe	AOD	-0.17	[-0.29 ; 0.04]	YES
UFaBr	MFaBr	0.1	[0.01 ; 0.19]	NO
UFaBr	PmBr	0.17	[0.05 ; 0.33]	NO
UFaBr	FaHe	-0.04	[-0.13 ; 0.08]	YES
UFaBr	AOD	-0.07	[-0.23 ; 0.07]	YES
MFaBr	PmBr	0.09	[-0.04 ; 0.24]	YES
MFaBr	FaHe	-0.13	[-0.23 ; 0.01]	YES
MFaBr	AOD	-0.18	[-0.32 ; -0.02]	NO
PmBr	FaHe	-0.22	[-0.35 ; -0.06]	NO
PmBr	AOD	-0.28	[-0.42 ; -0.10]	NO
FaHe	AOD	-0.05	[-0.21 ; 0.10]	YES

Supplementary Table S.3. Posterior modes and 95% highest posterior density (HPD) intervals of pairwise differences in evolvability (*e*) among traits. For each pair of traits, we computed the posterior distribution of the difference by subtracting posterior samples of evolvability obtained from the fitted Bayesian mixed model (model 9, see main manuscript for evolvability equation). Each difference distribution is summarized by its posterior mode and 95% highest posterior density (HPD) interval. A difference is considered credibly different from zero when its 95% HPD interval does not include zero. Abbreviations: FaLe – Facial length; NaLe – Nasal length; PmLaLe – Premaxilla lateral length; PmLe – Premaxilla length; UFaBr – Upper facial breadth; MFaBr – Mid-facial breadth; PmBr – Premaxilla breadth; FaHe – Facial height.

		Posterior distribution of the difference		
**Trait 1**	**Trait 2**	**Mode**	**95% HPD**	**Include 0**
FaLe	NaLe	-0.00139	[-0.00226 ; -0.00070]	NO
FaLe	PmLaLe	0.00033	[-0.00141 ; 0.00126]	YES
FaLe	PmLe	-0.00188	[-0.00311 ; -0.00081]	NO
FaLe	UFaBr	0.00191	[0.00112 ; 0.00259]	NO
FaLe	MFaBr	0.00198	[0.00119 ; 0.00271]	NO
FaLe	PmBr	0.00038	[-0.00091 ; 0.00150]	YES
FaLe	FaHe	0.00082	[0.00032 ; 0.00155]	NO
FaLe	AOD	0.00297	[0.00207 ; 0.00351]	NO
NaLe	PmLaLe	0.00156	[-0.00014 ; 0.00305]	YES
NaLe	PmLe	-0.00026	[-0.00202 ; 0.00126]	YES
NaLe	UFaBr	0.00331	[0.00235 ; 0.00441]	NO
NaLe	MFaBr	0.00346	[0.00254 ; 0.00462]	NO
NaLe	PmBr	0.00207	[0.00033 ; 0.00314]	NO
NaLe	FaHe	0.00247	[0.00156 ; 0.00334]	NO
NaLe	AOD	0.00447	[0.00339 ; 0.00532]	NO
PmLaLe	PmLe	-0.00168	[-0.00347 ; -0.00037]	NO
PmLaLe	UFaBr	0.00163	[0.00068 ; 0.00319]	NO
PmLaLe	MFaBr	0.00159	[0.00086 ; 0.00329]	NO
PmLaLe	PmBr	0.00032	[-0.00058 ; 0.00130]	YES
PmLaLe	FaHe	0.00079	[-0.00029 ; 0.00231]	YES
PmLaLe	AOD	0.00299	[0.00154 ; 0.00405]	NO
PmLe	UFaBr	0.00375	[0.00239 ; 0.00511]	NO
PmLe	MFaBr	0.0036	[0.00253 ; 0.00521]	NO
PmLe	PmBr	0.00215	[0.00053 ; 0.00364]	NO
PmLe	FaHe	0.00288	[0.00159 ; 0.00424]	NO
PmLe	AOD	0.00475	[0.00350 ; 0.00620]	NO
UFaBr	MFaBr	0.00008	[-0.00018 ; 0.00042]	YES
UFaBr	PmBr	-0.00168	[-0.00261 ; -0.00056]	NO
UFaBr	FaHe	-0.00088	[-0.00140 ; -0.00046]	NO
UFaBr	AOD	0.00092	[0.00066 ; 0.00126]	NO
MFaBr	PmBr	-0.00163	[-0.00266 ; -0.00063]	NO
MFaBr	FaHe	-0.00107	[-0.00158 ; -0.00052]	NO
MFaBr	AOD	0.0009	[0.00051 ; 0.00115]	NO
PmBr	FaHe	0.00064	[-0.00038 ; 0.00181]	YES
PmBr	AOD	0.00241	[0.00153 ; 0.00357]	NO
FaHe	AOD	0.00179	[0.00137 ; 0.00241]	NO
